# SEMA3A and IGSF10 Are Novel Contributors to Combined Pituitary Hormone Deficiency (CPHD)

**DOI:** 10.3389/fendo.2020.00368

**Published:** 2020-06-16

**Authors:** Bartlomiej Budny, Tomasz Zemojtel, Malgorzata Kaluzna, Pawel Gut, Marek Niedziela, Monika Obara-Moszynska, Barbara Rabska-Pietrzak, Katarzyna Karmelita-Katulska, Marek Stajgis, Urszula Ambroziak, Tomasz Bednarczuk, Elzbieta Wrotkowska, Ewelina Bukowska-Olech, Aleksander Jamsheer, Marek Ruchala, Katarzyna Ziemnicka

**Affiliations:** ^1^Department of Endocrinology, Metabolism and Internal Diseases, Poznan University of Medical Sciences, Poznan, Poland; ^2^Institute for Medical and Human Genetics, Charité-Universitätsmedizin Berlin, Berlin, Germany; ^3^Genomics Platform, Berlin Institute of Health, Berlin, Germany; ^4^Department of Pediatric Endocrinology and Rheumatology, Poznan University of Medical Sciences, Poznan, Poland; ^5^Department of General Radiology and Neuroradiology, Poznan University of Medical Sciences, Poznan, Poland; ^6^Department of Internal Medicine and Endocrinology, Medical University of Warsaw, Warsaw, Poland; ^7^Department of Medical Genetics, Poznan University of Medical Sciences, Poznan, Poland

**Keywords:** pituitary, CPHD, *PROP1*, *SEMA3A*, *IGSF10*, *CHD7*

## Abstract

**Background:** The mutation frequencies of pituitary transcription factors genes in patients with combined pituitary hormone deficiencies (CPHD) vary substantially between populations. However, apart from *PROP1* the mutation rate of other genes is low and for almost half of the patients with CPHD the routine sequencing of known genes is unsuccessful in the identification of genetic causes.

**Methods:** A cohort of 66 sporadic and nine familial CPHD cases (80 patients in total) were subjected to initial testing of the genes *PROP1, POU1F1, LHX3, LHX4*, and *HESX1* using a targeted gene panel and MLPA. In patients who tested negative, a whole exome sequencing approach was employed.

**Results:** In nine of the familial cases and 32 of the sporadic patients mutations in the *PROP1* gene were found (the common pathogenic variants included c.301_302delAG and c.150delA). Mutations were also found in genes so far not related directly to CPHD. A unique homozygous and clinically relevant variant was identified in the *SEMA3A* gene, which may contribute to neural development and his phenotypic spectrum including short stature and isolated hypogonadotropic hypogonadism (IHH). Another pathogenic variant p.A1672T was found in the *IGSF10* gene reported to be responsible for delayed puberty and neuronal migration during embryogenesis. Several suspected novel but predicted benign variants were also identified for the *CHD7, WDR11* and *FGF17* genes.

**Conclusion:** Although *PROP1* defects account for a majority of CPHD patients, identification of rare, less frequent variants constitutes a big challenge. Multiple genetic factors responsible for CPHD are still awaiting discovery and therefore the usage of efficient genomic tools (i.e., whole exome sequencing) will further broaden our knowledge regarding pituitary development and function.

## Introduction

Combined Pituitary Hormone Deficiency (CPHD) is caused by both genetic and nongenetic factors including trauma, tumor, infection, irradiation, and autoimmune diseases. The prevalence of CPHD was recently estimated to be 1 in 8,000 individuals worldwide (Genetics Home Reference at NIH, https://www.ghr.nlm.nih.gov). The genetic defects causing CPHD typically result in misdevelopment of the anterior pituitary gland and insufficient hormone secretion, which manifests in early childhood. So far ~30 genes have been found to cause CPHD in its nonsyndromic or more severe syndromic forms (1). The most frequently mutated genes belong to the transcription factors family including PROP1, HESX1, LHX3, LHX4, POU1F1, GLI2, OTX2 but mutations within those genes would explain only some cases (2). The entire picture of pituitary hormone deficiency is furthermore emphasized by its associated severe syndromic phenotypes including craniofacial defects i.e., septo-optic dysplasia SOD (*HESX1, OTX2, GLI2, GLI3*) or holoprosencephaly (*SHH*). Recently, the phenotypic spectrum of CPHD has broadened due to the reported overlapping manifestation with isolated hypogonadotropic hypogonadism (IHH). This was somewhat expected since both conditions may share similar etiologies. The co-occurrence of IHH in CPHD phenotype was already well proven in regards to the *GLI2, SOX2*, and *SOX3* genes and recently pituitary dysfunction was confirmed for well characterized IHH genes namely CHD7, PROKR2, FGFR1, and FGF8 (3). The recent improvement of genetic testing technologies and the possibility of screening all genes within a patient should quickly be beneficial in broadening the list of CPHD-IHH overlapping phenotypes, particularly for the gonadotropin related genes but also others contributing to the hypothalamic-pituitary axis.

This study reports a comprehensive genetic examination of participating CPHD patients to identify defects in previously known genes as well as genes at new loci in order to recognize novel genetic mechanisms of the development of the CPHD.

## Patients

The present study included a total of 80 patients with CPHD. All participating patients were diagnosed according to standard procedures in the Department of Pediatric Endocrinology and Rheumatology, in the Department of Endocrinology, Metabolism and Internal Diseases at Poznan University of Medical Sciences and in the Department of Internal Medicine and Endocrinology, Medical University of Warsaw. Among these patients were 66 sporadic patients and nine familial cases of the disease (14 affected individuals in total). An average age at the moment of diagnosis was 9.2 ± 6.1 years. All adult patients at the time of the study were retested in adulthood. All of these patients suffered from GH deficiency, 98.8% had gonadotropins and TSH deficiency, 32.5% had prolactin deficiency and 33.8% had ACTH deficiency. Pituitary Magnetic Resonance Imaging (MRI) was performed in all patients. Methods describing evaluation of hormonal status and pituitary imaging were described in our previous publication (4). In familial cases of the disease, no consanguinity was reported for any of the recruited families. All subjects were informed about the purpose of the study and their written consent was obtained. The Bioethical Committee of Poznan University of Medical Sciences approved the study. A population cohort of 104 individuals with WES data, and originating from the same region of Poland was used as a control group for excluding population specific variants while using WES in CPHD patients.

## Genetic Examinations

### Targeted Sequencing of Pituitary Genes

In order to search for mutations in known genes involved in pituitary organogenesis, patients were routinely screened using a targeted panel for point mutations in five pituitary genes. The bi-directional capillary sequencing was employed for *PROP1, HESX, POU1F1, LHX3*, and *LHX4*, genes. For all selected genes the coding sequences, together with ~50 nucleotides from neighboring intronic regions, were amplified from genomic DNA in independent PCR reactions [conditions were already published (5)]. Primers were designed using the Primer3 program (http://www.genome.wi.mit.edu/genome_software/other/primer3.html/). The PCR products were visualized on a 2% agarose gel and purified using an Affymetrix ExoSAP-IT enzymatic PCR clean-up system. Sequencing reactions were performed using the BigDye Terminator kit (PE Biosystems, Rockville, USA) and subsequently visualized using a 3730xl DNA Analyzer (Applied Biosystems, Rockville, USA). The sequence tracks were further evaluated for quality metrics and subsequently examined for mutations with the use of CodonCode Aligner (http://www.codoncode.com), and reference sequence retrieved from GenBank (http://www.ncbi.nlm.nih.gov/genbank).

### Whole Exome Sequencing and Pathogenicity Prediction

Patients with excluded mutations in *PROP1, HESX, POU1F1, LHX3*, and *LHX4* genes were analyzed further using exome sequencing. A total of 1 μg of genomic DNA from subjects was used for the construction of a library with the TruSeq DNA Sample Preparation Kit (Illumina). Whole exome enrichment was performed with the use of DNA library and TruSeq Exome Enrichment Kit (Illumina). The achieved assay performance was as follows: The minimal mean depth of target regions was 218x (post-alignment) and the average read length 149 bp. The percentage of bases in target regions with a depth of coverage >20X−98.6%, >30X−97.5%, >50x−94.1%. Ninety-nine percentage of the reads was aligned and mapped to human genome reference sequence hg19 (BWA v0.7.12, Picard v1.130, GATK v3.4.0, SnpEff v4.1g) of which 90.5% were non-redundant. For pathogenicity evaluation, the following features were applied: (a) gene/transcript annotations (downloaded from UCSC GenomeBrowser, hg19), (b) known sequence variants from dbSNP (version 142), 1,000 Genomes project (The 1,000 Genomes Project Consortium, Phase3), ExAc Database (http://exac.broadinstitute.org/) and GenomAD v2.1.1 (6). Selected alterations, which were found using NGS were confirmed using Sanger sequencing and no discrepancy between these results was detected. The identified variants were analyzed for their impact on protein structure and functionality and pathogenicity estimation using following algorithms: Sift (7), and PolyPhen2 (8), dbNSFP (9), FATHMM (10), MutationTaster v2 (11), PhenIX (12), CADD (13), and HPO database (14) as well as population datasets. Nucleotide sequences (coding and neighboring ~50 nt intronic) were explored for Splice Site disturbances using Human Splicing Finder (HSF3.0) (15) and NNsplice v. 0.9 (16). The variant prioritization was based on the variant frequency (novel or very rare variants), functional prediction using selected algorithms (VEP tool, Ensembl), and possible contribution of a genes into pituitary functioning (HPO) (17). All variants were classified according ACMG recommendations (18) as presented in **Table 2**, with the use of InterVar tool (19) and Varsome (20), checked individually for correctness of estimation and adjusted manually if needed. Prediction of protein conformation changes was accomplished using Phyre2 (21) and UCSF Chimera 1.7 (22).

## Results

Among examined CPHD patients a subtle predominance of males (43M/37F) was noted that is in accordance with published epidemiologic data (23–25). Abnormalities in pituitary morphology were observed in majority of patients (95%). Pituitary Stalk Interruption Syndrome (PSIS) was diagnosed in 12 patients (22.7%). The analysis of major CPHD transcription factors resulted in identification of mutations in all nine familial cases (14 patients) and 32 sporadic patients (45% of all patients), all without PSIS. Mutations were found only in *PROP1* gene and they represented recessive founder 2-bp deletion c.301_302delAG (p.L102Cfs^*^8) and c.150delA (p.R53Dfs^*^112). Among these mutations, homozygosity for c.301_302delAG was detected in 11 cases (30% of patients affected by *PROP1* mutation), homozygosity for c.150delA in 10 patients (27%) and 15 cases presenting compound heterozygosity (41%). No abnormalities were identified in *HESX, POU1F1, LHX3*, and *LHX4* genes in the coding sequences. Neither structural rearrangements within those genes, nor abnormalities in particular exon copy number were detected using MLPA (unpublished data). Clinical characteristics of the patients regarding their genetic status was reported in Table 1.

**Table 1:** Clinical characteristics of studied CPHD patients in regard to genetic status (patients with mutations in other genes are presented in Table 3).

**Mutation status**	**No of patients**	**Gender (F/M)**	**Age at diagnosis (mean ±- SD)**	**Pituitary hormone deficiency**					**MRI of pituitary**
				**GH**	**GnRH**	**TSH**	**PRL**	**ACTH**	
*PROP1* gene mutation (total)	36	19/17	7.4 (±6,4)	36 (100%)	36 (100%)	36 (100%)	22 (61%)	8 (22%)	Normal pituitary - 3 Hypoplasia - 33 PSIS - 0
*PROP1* c.[301_30‘2delAG]; [301_302delAG]	11	5/6	7.5 (+6.3)	11 (100%)	11 (100%)	11 (100%)	7 (64%)	2 (18%)	Normal pituitary - 0 Hypoplasia - 11 PSIS - 0
*PROP1* c.[150delA]; [c.150delA]	10	7/3	7.7 (±6.8)	10 (100%)	10 (100%)	10 (100%)	6 (60%)	2 (20%)	Normal pituitary - 1 Hypoplasia - 9 PSIS - 0 m
*PROP1* c.[150delA]; [301_302delAG]	15	6/9	7.1 (±6.1)	15 (100%)	15 (100%)	15 (100%)	9 (60%)	4 (27%)	Normal pituitary - 2 Hypoplasia - 13 PSIS - 0
Patients without mutation	35	14/21	10.5 (±5.1)	35 (100%)	35 (100%)	35 (100%)	4 (11.4%)	16 (45.7%)	Normal pituitary - 0 Hypoplasia - 23 PSIS - 12

### Whole Exome Sequencing in PROP1 Negative Patients

**Figure 1:**
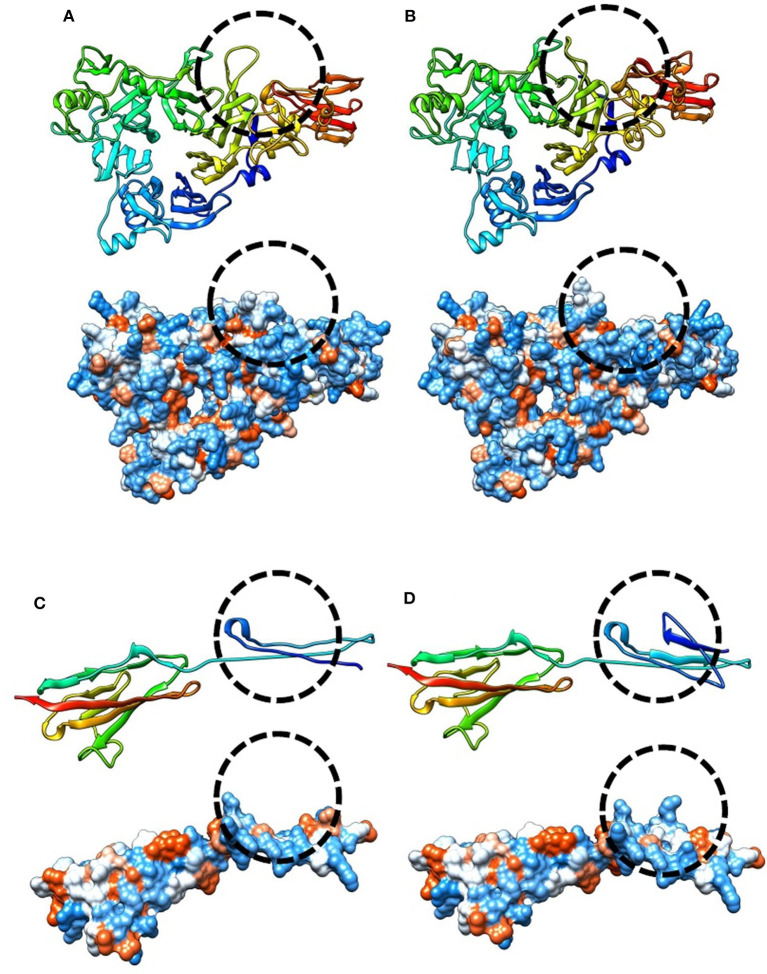
Modeling and prediction of conformational changes of SEMA3A and IGSF10 variants. Predicted conformation changes surrounding variant position are highlighted and encircled using dashed line. **(A)** Ribbon (top) and hydrophobic (bottom) model of reference SEMA3A protein sequence, **(B)** Ribbon (top) and hydrophobic (bottom) prediction of SEMA3A p.V435I variant. **(C)** Ribbon (top) and hydrophobic (bottom) model of reference IGSF10 sequence covering a third Ig-like C2-type domain (1648-1739AA), **(D)** Ribbon (top) and hydrophobic (bottom) prediction of p.A1672T variant affecting third Ig-like C2-type domain of IGSF10 protein.

All of the patients that did not reveal any abnormalities in conventional sequencing of the five most common CPHD genes were subjected for whole exome sequencing (WES). In two cases, likely pathogenic (according to ACMG criteria) and protein damaging variants affecting *IGSF10* and *SEMA3A* genes were identified. For the *IGSF10* gene a novel variant c.5014G>A: p.A1672T was found (Case 1). Analysis of relatives revealed *de novo* origin of this alteration. In another patient presenting CPHD with PSIS (Case 3), a unique likely pathogenic, homozygous *SEMA3A* allele carrying two variants: c.1302_1303delinsCA (p.V435delinsI) and c.1453-9delC was detected. The first reported variant c.1302_1303delinsCA is novel and likely pathogenic, whereas the second is rare (MAF 0.01) and therefore considered as benign with a rather minor impact. For these two patients and their detected variants, all used pathogenicity prediction algorithms consistently showed deleterious effect (Table 2, Figure 1), supporting causativeness.

**Table 2:** Exome sequencing results of studied patients with CPHD including variant description, effect on protein, allele frequency, classification and pathogenicity prediction.

**Variants predicted to affect protein function (pathogenic/VUS according to ACMG criteria)**											
**Case**	**Gene**	**Chrom. Position hg19**	**Variant description HGVS, nucleotide, protein, zygosity**	**Effect on protein, *in silico* prediction**	**dbSNP (rs number)**	**Mode of inheritance**	**ACMG variant classification**	**ACMG evidence**	**MAF gnomAD v2.1.1**	**CADD Score PHRED/RAW**	**Protein function**
1	IGSF10	3:151162755	p.A1672T, c.5014G>A, NM_178822.4 het.	Damaging, conformation change	Novel	Unknown (AD postulated)	Likely pathogenic	PS2, PM1, PM2, PP4,	–	33/6.91	Control of early migration of neurons expressing gonadotropin-releasing hormone (GnRH neurons)
2	IGSF10	3:151154509	p.D2614N c.7840G>A, NM_178822.4 het.	Damaging, conformation change	rs112889898	Unknown (AD postulated)	VUS/Benign	PM2, PM5	0.007	28.5/6.15	As above
	GLI2	2:121748048 2:121747544	p.D1520N(:)M1352V c.4558G>A(:)c.4054A>G comp.het.	Probably damaging	rs114814747 rs149140724	AD	VUS/Benign	PM2 PM5	0.009 0.010	32/4.80 7.8/0.55	Transcription factor which bind DNA through zinc finger motifs, mediator of Sonic hedgehog (Shh) signaling
3	SEMA3A	7:83634712-83634713	p.V435delinsI, c.1302_1303delinsCA NM_006080 hom.	Damaging, conformation change,	Novel	AD	Likely pathogenic	PM1, PM2, PM6, PP4,	–	26.6/3.82	Chemorepulsive factor, inhibiting axonal outgrowth, and chemoattractive agent, stimulating the growth of apical dendrites
		7:83614801-83614802	c.1453-9del C NM_006080 hom.	Splicing affected, acceptor gained	rs141423527	AD	Benign	PP3, PP4, BP7	0.01	10.74/0.65	
**Variants predicted to affect splicing (VUS/benign according to ACMG criteria)**											
4	IGSF10	3:151161101	p.L1878L, c.5634C>G, NM_178822.4 het.	Synonymous SNV, splicing affected, activation of an exonic cryptic donor site	Novel	Unknown (AD postulated)	VUS Uncertain significance	PS4, PM2, PM6, PP3, BP4, BP7,	–	5.84/0.20	Control of early migration of neurons expressing gonadotropin-releasing hormone (GnRH neurons)
	OTX2	14:57268750	p.G199G c.597C>G, NM_001270525.1, het.	Synonymous SNV, splicing affected, activation of an exonic cryptic donor site	Novel	AD	Benign	PM2, PM6, PP3, PP4, BP4, BP7	–	20.8/2.14	Transcription factor that plays a role in brain, craniofacial, and sensory organ development. Mutations in this gene cause syndromic microphthalmia 5 (MCOPS5) and combined pituitary hormone deficiency 6 (CPHD6)
5	SEMA3A	7:83764113	p.Q89Q, c.267A>G NM_006080 het.	Synonymous SNV, splicing affected, donor lost	rs74349534	AD	Benign	PP4, BS1, BS2, BP4, BP7,	0.009	15.94/1.53	Chemorepulsive factor, inhibiting axonal outgrowth, and chemoattractive agent, stimulating the growth of apical dendrites
	PCSK1	5:95757703	c.544-43T>G NM_000439.4	Splicing affected, acceptor gained	Novel	AR	Benign	PM6, PP4, BS1, BS2, BP4, BP7,	–	1.74/0.002	Proteolytic activation of polypeptide hormones and neuropeptides precursors
6	CHD7	8:61655329	p.G446G, c.1338A>G NM_017780 het.	Synonymous SNV, splicing affected, enhancer site broken	Novel	AD	VUS Uncertain significance	PS4, PM2, PM6, PP4, BP4, BP7,	–	15.78/1.51	Transcription regulator, commonly mutated in hypogonadotropic hypogonadism patients
7	WDR11	10:122622238	c.527-9T>C NM_018117.11 het.	Intron, splicing affected acceptor gained	Novel	AD	Benign	PM6, PP4, BS1, BS2, BP4, BP7,	–	18/1.89	Involved in a variety of cellular processes, including cell cycle progression, signal transduction, apoptosis, and gene regulation
8	FGF17	8:21900402	c.-38delG NM_003867.3 het.	5'UTR, splicing affected, donor gained	rs147561706	AD	Benign	PM6, PP4, BS1, BS2, BP4, BP7,	0.009	17.56/1.81	Essential for vascular growth and normal brain development. Mutations in this gene are the cause of hypogonadotropic hypogonadism 20 with or without anosmia
	FGF8	10:103530438	c.445-62G>A NM_033163.3, het.	Splicing affected, acceptor gained	rs3218238	AD	Benign	PM6, PP4, BS1, BS2, BP4, BP7,	0.02	9.18/0.71	Variety of biological processes, including embryonic development, cell growth, morphogenesis, tissue repair. Supports androgen and anchorage independent growth of mammary tumor cells
9	GLI2	2:121742124	p.T587T; c.1761G>A NM_005270.4, het.	Synonymous SNV, splicing affected, activation of an exonic cryptic acceptor site, with presence of one or more cryptic branch points	rs61732852	AD	Likely Benign	PM2, BP4, BP7,	0.004	2.3/−0.01	Transcription factor which bind DNA through zinc finger motifs, mediator of Sonic hedgehog (Shh) signaling

**Figure 2:**
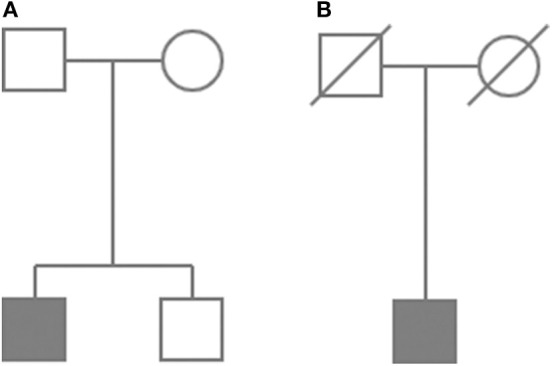
Abbreviated pedigrees of Case 1 **(A)** and Case 3 **(B)**. Symbols indicate: square for males and circle for females. Affected individuals are depicted as a blackened symbols. Diagonal line shows deceased individuals.

Except for these two cases, less convincing evidence and clinical relevancy was found for the detected variants in another seven patients (classified as variants of unknown significance VUS or benign according to ACMG criteria). For Case 2 (the patient presenting CPHD with PSIS), the damaging prediction was established for c.7840G>A (p.D2614N; rs112889898) of *IGSF10* and the two coexisting variants in CPHD gene *GLI2* (rs114814747, rs149140724). However, these changes were considered as VUS, since they were reported in population databases. In another patient (Case 4), synonymous change in *IGSF10* (c.5634C>G; p.L1878L) co-occurred with alteration in *OTX2* (c.597C>G; p.G199G), another gene with evidenced contribution to CPHD. These changes represent novel unreported variants, but only splice-site algorithms supported estimation of pathogenicity and therefore further functional experiments are required. Pathogenic, disease-causing splice-site predictions were also found for another five variants. In *SEMA3A* gene, a splice variant c.267A>G (p.Q89Q) was found in combination with heterozygous intronic change in the *PCSK1* gene (c.544-43T>G). Novel variants in two genes linked to hypogonadotropic hypogonadism: *CHD7* (c.1338A>G, p.G446G) and *WDR* (c.527-9T>C, intronic) were identified in the next two patients. In total four out of nine cases we noted co-occurrence of predicted deleterious variants in two different genes, supporting growing recent evidence on possible oligogenic background of CPHD. All changes with their genomic coordinates and pathogenicity prediction were included in Table 2. All of the novel variants were screened in the population cohort (104 controls, 208 alleles) and were absent. The clinical characteristics of patients affected by mutations in genes excluding *PROP1*, are presented in Table 3. It is noteworthy that four of the detected mutations were identified in subjects presenting CPHD with PSIS and five other CPHD without PSIS. The protein conformation prediction for *IGSF10* p.A1672T variant (Case 1) and *SEMA3A* p.V435delinsI variant (Case 3) are shown in Figure 1. Pedigrees for both cases are presented in Figure 2.

**Table 3:** Clinical characteristics of studied CPHD patients with genetic defects in *SEMA3A, IGSF10, PCSK1*, and IHH genes.

**Case**	**Gene/variant**	**Gender**	**Age at diagnosis**	**Pituitary hormone deficiency**					**MRI of pituitary**
				**GH**	**Gn**	**TSH**	**PRL**	**ACTH**	
1	**IGSF10**, p.A1672T c.5014G>A	M	13 y.o.	+	+	+	–	–	Pituitary hypoplasia
2	**IGSF10**, p.D2614N, c.7840G>A	F	6 y.o.	+	+	+	–	–	PSIS
	**GLI2**, p.D1520N/p.M1352V								
3	**SEMA3A**, c.1302_1303delinsCA:p.V435delinsI	M	9 y.o.	+	+	+	–	+	PSIS
	c.1453-9del								
4	**IGSF10**, p.L1878L,c.5634C>G	F	31 y.o.	+	–	–	–	+	Pituitary hypoplasia
	**OTX2**, p.G199G, c.597C>G								
5	**SEMA3**, p.Q89Q c.267A>G	M	15 y.o.	+	+	+	–	+	PSIS
	**PCSK1**, c.544-43T>G								
6	**CHD7**, p.G446G c.1338A>G	F	8 y.o.	+	+	–	–	–	PSIS
7	**WDR11**, c.527-9T>C	M	7 y.o.	+	+	+	–	–	Pituitary hypoplasia
8	**FGF17**, c.-38delG	M	12 y.o.	+	+	+	–	–	Pituitary hypoplasia
	**FGF8**, c.445-62G>A								
9	**GLI2**, p.T587T; c.1761G>A	F	8 y.o.	+	+	+	–	–	Pituitary hypoplasia

*In all reported patients with new genetic defects, no other clinical abnormalities were reported*.

## Discussion

**Figure 3:**
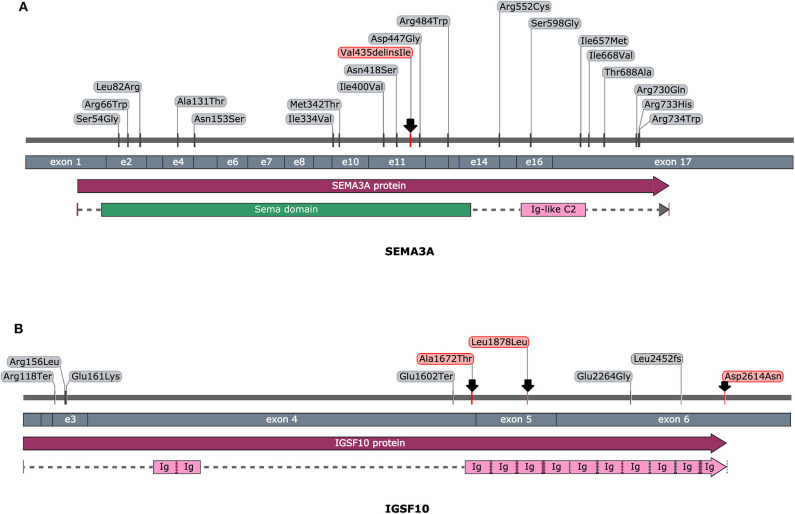
An overview of reported pathogenic variants in *SEMA3A* **(A)** and *IGSF10* **(B)** gene, according to HGMD database. Variants are ordered in regard of nucleotide coding position (top section), exonic location (middle section) and protein sequence with highlighted functional domains (bottom section). Variants identified in this study were depicted with an arrow. Picture was prepared with the use of SnapGene software (GSL Biotech, snapgene.com).

In this study we examined 80 CPHD patients (nine families, 66 sporadic cases), searching for genetic abnormalities. Causative mutations were found in the *PROP1* transcription factor gene as well as two other genes known to contribute to isolated hypogonadotropic hypogonadism (IHH), namely *IGSF10* and *SEMA3A*. Our results are in agreement with multiple studies reporting the occurrence of common *PROP1* founder mutations in sporadic and familial cases (26–29). The most frequent mutation in our cohort was the 2-bp deletion c.301_302delAG (p.Leu102Cysfs^*^8), detected in 26 cases (11 homozygotes and 15 compound heterozygotes). This frameshift deletion is the most common *PROP1* mutation and leads to a premature stop codon in the residue 110. The second most common *PROP1* variant was 1-bp deletion c.150delA (p.Arg53Aspfs^*^112) found in 25 cases (10 homozygotes and 15 compound heterozygotes). The high frequency of both mutations is caused due to the presence of two ancestral founder variants - c.301_302delAG originated from the Baltic Sea area around 2,525 years ago and c.150delA from the Belarus region around 1,093 years ago (30, 31). There were no other changes reported for this gene, which is not surprising since other mutations are ultra-rare and contribute to a minority of patients (5, 32). The other disruptive variants affecting *POU1F1, LHX3*, and *LHX4*, contribute only occasionally to the pituitary phenotype (33–35). In this study, we also detected likely pathogenic occurrence of point mutations disturbing the *SEMA3A* and *IGSF10* genes, so far not linked to CPHD. The semaphorin 3A gene (*SEMA3A*) is a member of the semaphorin family and encodes a protein composed of immunoglobulin-like domain (C2-type), a PSI domain and major Sema domain. This protein shows activity as either a chemorepulsive agent, inhibiting axonal outgrowth, or as a chemoattractive agent, stimulating the growth of apical dendrites (36). In both cases, the protein is vital for normal neuronal pattern development. Semaphorin 3 induces the collapse and paralysis of neuronal growth cones and serves as a ligand that guides specific growth cones by a motility-inhibiting mechanism (37). The protein is therefore involved in the development of the olfactory system and in neuronal control during puberty. Due to *SEMA3A* genes‘ pivotal role in development, the gene was widely examined for contribution to various human disorders. Heterozygous variants have been associated with hypogonadotropic hypogonadism and anosmia (Kallmann syndrome, OMIM # 614897, autosomal dominant inheritance) (38–41). In another report Hofmann et al., presented biallelic *SEMA3A* loss of function mutations causing syndromic short stature (42). The phenotype of the reported patient resembles knock-out mice symptoms (relative macrocephaly, camptodactyly, septal heart defect) indicating a novel autosomal recessive type of syndromic short stature without obvious signs of Kallmann syndrome. In this report, we found for the first time *SEMA3A* allele bearing two variants (c.1302_1303delinsCA, c.1453-9delC) in the homozygous state, thus indicating a novel attractive target contributing to pituitary organogenesis. The identified variant is supporting evidence of recessive inheritance and milder outcome comparing to previously reported conditions. Another likely pathogenic variant was found in the *IGSF10* gene, which is a player recently evidenced to be causative for isolated hypogonadotropic hypogonadism and delayed puberty. Immunoglobulin Superfamily Member 10 (IGSF10) is a component of a complex system of migratory cues guiding GnRH neurons from their origin in the nasal placode toward the hypothalamus during embryogenesis. Howard et al. showed that *IGSF10* mutations dysregulate gonadotropin-releasing hormone neuronal migration (43). Using exome and candidate gene sequencing, the authors identified rare heterozygous mutations in IGSF10 in six unrelated families, which resulted in intracellular retention with failure in the secretion of mutant proteins and self-limited delayed puberty segregated within families, displaying an autosomal dominant pattern of inheritance. A pathogenic variant in IGSF10 was found in one of our CPHD patients. Another two patients presented with VUS/benign IGSF10 alterations that co-occurred with a variant in the CPHD gene (*GLI2* or *OTX2*) and, therefore, its true contribution to the disease is unclear. In addition to this, we also noted that all reported IGSF10 variants affect highly conserved residues among homologs, as revealed by PhyloP (an overview presenting reported mutations in IGSF10 and SEMA3A was shown in Figure 3). A recent report presenting WES examination in pituitary stalk interruption syndrome (PSIS), was published by Zwaveling-Soonwala et.al. (44). Authors indicated a particularly common contribution of rare *GLI2* variants for PSIS patients (in total 16 were reported). We identified *GLI2* rare change only for one out of 12 cases with PSIS - showing combined p.D1520N/p.M1352V changes (co-occurring with *IGSF10* p.D2614N) and in the other patient with pituitary hypoplasia without PSIS (a benign synonymous SNV p.T587T as well as predicted activation of the exonic cryptic splice-site). In our cohort of patients, however, we could not confirm presence of cadherin gene *DCHS1* defects frequently mutated in PSIS patients. It is noteworthy that among PSIS cases two other benign variants, in IHH genes *CHD7* and *FGF8*, were also identified (both predicted to affect splicing). These findings may support the growing role of IHH genes in etiopathogenesis of CPHD. In another study Simm et al. (45) reported two new genes contributing to CPHD, *SLC20A1*, and *SLC15A4* and evidenced that a significant portion of cases arise as a *de novo* event. Another study utilizing NGS sequencing also shed a new light on the combination of rare variants in different genes that might explain the incomplete penetrance in CPHD and the complexity of the disorder (46, 47). In conclusion, we confirmed the major contribution of *PROP1* gene mutations in CPHD. The analysis indicated founder deleterious variants as the most frequent changes present but no novel mutations were found. No other convincingly pathogenic changes were identified in other known CPHD transcription factors. Only the *GLI2* combined variant (p.D1520N/p.M1352V) was found in one patient which is seemingly more frequent among CPHD patients, as reported by Zwaveling-Soonwala et al. (44). Despite this, we identified a single pathogenic mutation in the *SEMA3A*, and *IGSF10* genes producing two new candidates potentially causative for CPHD etiology. Both genes are coding proteins that are involved in neuronal growth and migration, therefore potentially influencing proper pituitary development.

## Data Availability Statement

The datasets generated for this study can be found in the Department of Endocrinology, Metabolism and Internal Diseases, Poznan University of Medical Sciences, Poznan, Poland.

## Ethics Statement

The studies involving human participants were reviewed and approved by The Bioethical Committee of Poznan University of Medical Sciences. The patients/participants provided their written informed consent to participate in this study.

## Author Contributions

BB and KZ contributed to conception and design of the study. BB, TZ, EB-O, and AJ contributed to genomic analyses. KZ, MK, PG, MN, MO-M, BR-P, KK-K, MS, UA, TB, and MR performed clinical examinations. EW participate in lab work. BB and KZ wrote the first draft of the manuscript. All authors contributed to manuscript revision, read, and approved the submitted version.

## Conflict of Interest

The authors declare that the research was conducted in the absence of any commercial or financial relationships that could be construed as a potential conflict of interest.
